# Book Reviews

**Published:** 1917-06

**Authors:** 


### BOOK REVIEWS

Books mentioned may be inspected at and ordered through this office. So far as possible, books received in any month will be reviewed in the issue of the second month following. Pamphlets, quarterly and similar periodicals, reports, transactions, etc., will, as a rule, merely be mentioned.

**Zone Therapy or Relieving Pain at Home.** Wm. H. FitzGerald and Edwin F. Bowers, M. D., published by I. W. Long, Columbus, O. 191 pages, $1.50.

This is a most remarkable claim for the analgesic effects of pressure, variously applied, digitally, manually, by rubber bands, clothes pins and the like.

**Transactions .of the American Gastro-Enterological Assn** 19th meeting, Washington, May 8 and 9, 1916. Printed for the Assn. under direction of Dr. Horace W. Soper, St. Louis.

**How to Run an Automobile.** Victor W. Page, M. E., published by Norman W. Henley Publishing Co., 132 Nassau St., N. Y. 178 Pages, illustrated, $1.00.

This is divided in four parts: Automobile Parts and their Functions; General Starting and Driving Instructions; Typical 1917 Control Systems; Care of Automobiles. Between the colossal ignorance of salesmen and the lack of time and professional reticence of service men, it is very difficult for the owner of an automobile to get an intelligent idea of its anatomy, physiology and pathology. This book supplies the need as well as possible, short of practical demonstration in a school.

**Cancer, Its Cause and Treatment.** L. Duncan Bulkley, A. M., M. D., New York, published by Paul B. Hoeber, N. Y. 282 pages, $1.50.

This is designated as Volume (2 acorns) indicating that it is really a much enlarged edition of a previous work on the same subject. The aim of the author is to consider the problem broadly, by getting at the real nature of cancer, instead of emphasizing any single method of attack or indulging in pessimism on a statistic basis. That he does not succeed in stating either the essential cause, method of prevention or of cure, in the ideal degree, is due to general lack of knowledge but we believe the solution of the problem is greatly simplified by its statement in such terms. Dr. Bulkley regards cancer as not wholly due to traumatic causes, not due to a microorganism, not contagious, not hereditary, not especially due to occupation, sex, race, climate, or any single cause of any kind.

---

**The Internal Secretions.** E. Gley, M. D., Paris; translated by Maurice Fishberg, M. D., N. Y. Published by Paul B. Hoeber, N. Y., 240 pages, $2.00.

The especial value of this book lies in its consideration of fundamental principles: the distinction between the two kinds of secretion, the distinctive characters and principal products of the internal secretions, while in considering the secretions in detail, the reciprocal relations are thoroughly discussed. The reader who looks for ''pointers'' either in diagnosis or therapeutics, will be disappointed and may even accuse the author of not being practical but, at this period, when the literature is crowded with itemized descriptions of hyperthyroidism, pituitary action, with reports of chemic and psysiologic experiments, nothing could be more valuable than a work of this nature which will help the student to correlate and to criticise along broad lines, the ordinary run of writing on internal secretions.

---

**An Old Frontier of France.** The Niagara Region and Adjacent Lakes under French Control, by Frank H. Severance of Buffalo. Published by Dodd, Mead & Co., N. Y.

This work constitutes the 20th volume of the Buffalo Historical Society's publications but is divided into two volumes, aggregating nearly 900 pages, illustrated and indexed. Both as to compilation and editorship and as to most of the historic research, it represents the work of the author, the Secretary of the Society. While its value depends to a large degree upon the presentation of original, contemporary reports and descriptions (often, of course, translated), it is not merely a collection of such documents but the author's life-long interest in the history of the Niagara Frontier has enabled him to assume a critical attitude and to arrange the material in an orderly manner, with a sharp discrimination between history and traditional misconceptions. The title calls attention to the fact that the subject is far removed from merely local interest. The events chronicled and political forces studied, have had far-reaching influences on the development of the western continent and even on the subsequent history of the whole world.

**Sur Les Tetanos Post Seriques.** Auguste Lumiere, reprint from the Annals of the Pasteur Institute, Jan. 1917. 19 pages, paper cover.

This study is based on tables showing 15 cases without trismus, 13 with slight or delayed trismus, 26 with trismus, with respectively 1, 3 and 17 deaths from tetanus and 3, 2 and 1 from intercurrent affections. 27 cases of tetanus without prophylactic injection, resulted in 19 deaths from tetanus and 1 from intercurrent affection. This subject is of great interest owing to the present war.

---

**The Problem of the Nations.** A Study of the Causes, Symptoms and Effects of Sexual Disease, and the Education of the Individual Therein. A. Corbett Smith, M. A., Barrister-at-Law, F. R. G. S., Editor of the Journal of State Medicine. Published by John Bale, Sons & Danielsson, Ltd., London. 107 pages, paper cover.

This monograph is based on statistics, of special value to American readers because of sources not usually included in American works on the subject. The point of view is sociologic rather than medical.

---

**Practical Treatise on Fractures and Dislocations.** Lewis A. Stimson, B. A., M. D., LL. D., New York, published by Lea & Febiger, N. Y. and Philadelphia. 8th edition, 946 pages, 475 illustrations and 39 monotint plates, $6.00.

The Hudson St. Hospital, N. Y., 1894-1905, cared for 14,556 fractures and 1527 dislocations. The distribution of the fractures was: cranium 5.5%; face and neck 8.79% (the great majority being of the nasal bones and lower jaw, there being only 3 of the hyoid and cvertebral fracture not being included here); 12.57% (about 12% of ribs alone); upper extremity 46.8% nearly 30% being included among Colles', metacarpal and phalangeal fractures; lower extremity 26.54%. Tables of seasonal incidence of fractures are given, which do not coincide very closely with a priori reasoning. It is scarcely necessary to say that the book is thorough and that X-ray diagnostics and the newer methods of treating fractures are properly considered. What impresses one is rather the fact that a subject is followed up to the end. Pathologic changes and unfortunate results are discussed so as to afford a timely warning. Statistics and notes of unusual cases are frequently introduced and the attempt is made throughout the work to give all possible useful information bearing on the treatment and outcome of fractures and dislocations.

---

**Rest, Suggestion and Other Therapeutic Measures in Nervous and Mental Diseases.** Francis X. Dercum, A. M., M. D., Ph. D., Philadelphia. P. Blakiston's Son & Co., Philadelphia. 2d edition, 395 pages, $3.50.

The chemic and physical changes in functionating organs, the essential nature of fatigue and allied fundamental problems are considered. The distinction between physiologic and therapeutic rest is well made and the relation of rest to exercise and the degrees of rest necessary in treatment are discussed in a particularly enlightening way. The only criticism that we can make in this regard is that some practitioners, even when prescribing rest for themselves, need an even more explicit emphasizing of the fact that rest is not inertia but recuperation and that many patients need a good time more than idleness. In some respects, the author's ideas as to diet may be considered as too strongly biased by neurologic specialization—too much milk and raw eggs. "white meat," etc. Many valuable suggestions are contained in the sections devoted to hysteria and hypochondria. Leading up to the control of nervous conditions by suggestion, there is a very interesting historic resume of mystic and religious methods and those of early charlatanry.

---

**A Compend of Human Physiology.** Albert P. Brubaker, A. M., M. D., Philadelphia, P. Blakiston's Son & Co. 14th edition, 262 pages, 26 illustrations, $1.25.

This is the original, brown covered, gilt lettered quiz compend which clinched the information that two generations of medical men have derived from lectures in medical colleges. It has been much though gradually changed since some of us first used it, to kep pace with advance in scientific discovery but it is the same old book in its essence. It isn't even "Brubaker's Compend, 14th edition by —— ——" like some old friends in recent editions and we hope that it will be many many editions more before that happens.

